# Analysis of the Correctness of Mapping the Passage of a Semi-Trailer through a Road Obstacle on a Road Simulator

**DOI:** 10.3390/s23198225

**Published:** 2023-10-02

**Authors:** Arkadiusz Czarnuch, Marek Stembalski, Tomasz Szydłowski, Damian Batory

**Affiliations:** 1 Department of Vehicles and Fundamentals of Machine Design, Lodz University of Technology, 90-924 Lodz, Poland; a.czarnuch@wielton.com.pl (A.C.); tomasz.szydlowski@p.lodz.pl (T.S.); damian.batory@p.lodz.pl (D.B.); 2 Wielton S.A., 98-300 Wieluń, Poland; 3 Department of Machine Tools and Mechanical Technologies, Wroclaw University of Technology, 50-370 Wroclaw, Poland

**Keywords:** sensors, road simulator, road load data, fatigue damage, durability test, vehicle

## Abstract

Road simulators enable accelerated durability tests under similar-to-real road conditions. However, the road simulator itself generates the signals with the appropriate strength and amplitude that is adequate to the response registered by the sensors during the real run. Therefore, there is a need for verification of the validity of the representation of vehicle runs on a road simulator in terms of the shape of the generated profile and possible sources of uncertainty. The tests in this study were carried out for a multi-axle vehicle passing an obstacle of known shape. Various signals were registered while the vehicle was passing over the obstacle. The MTS (System Corporation) road simulator’s response to the signal given by the obstacle was then checked. The results showed a 99% correlation between the simulation and the road test results. A numerical model of the vehicle was developed to verify the quality of representation of the real conditions by the road simulator, especially in terms of forces resulting from the road profile. Interestingly, the input signal generated by the road simulator provided a very good accuracy of the vehicle response, as tested with use of the numerical model.

## 1. Introduction

The transportation sector plays a vital role in the contemporary economy. According to Eurostat [1], the largest transport is maritime transport which holds a ca. of 67.9% of all cargo transport, followed by the road transport holding a ca. of 24.6%, while the rest is represented by railway and inland waterway transport (5.4% and 1.8%, respectively). Goods should be transported effectively, using adequate vehicles that ensure failure-free operation throughout their entire life. A common practice in the automotive sector is to use specialized road simulators to carry out durability tests of the vehicles being produced. The tests are aimed to represent the vehicle operating conditions as accurately as possible. In this way, the manufacturers can precisely determine the product’s reliability and lifetime. A process for establishing the vehicle durability test mode based on a user survey was presented by Fu [2].

Durability tests of an entire vehicle can be performed in different ways described in detail by Klyatis in [3]. Field tests in real conditions on roads of various quality reproduce the vehicle’s real behavior but entail an extended test duration and high costs. Another durability testing method involves vehicles driving on test tracks with artificially shaped surfaces to accelerate the tested vehicle’s wear over time. Kosobudzki et al. [4] analyzed a suspension stabilizer, as a selected vehicle component for durability testing. The authors determined a generalized durability index, d, used for comparing the durability of individual components. The generalized durability index d expresses numerically the overall impact of parameters describing the vehicle motion (e.g., speed and type of test section) on the durability of the component, but without reference to the material characteristics that the element is made of. Haiqiang et al. [5] presented a fatigue analysis of an engine bracket using a test track. The performed tests, durability calculations and microstructural analyses of the specimens helped the authors improve the bracket’s design. The final model of the bracket lasted more than twice the test section. The third durability testing method uses test rigs reproducing the real conditions or inputs (loads) from test tracks. The process of reproducing test track conditions on the MTS 329 road simulator for a passenger vehicle was described by Fricke et al. [6]. The presented methodology, based on Hybrid System Response Convergence, allowed the authors to combine physical and virtual simulation elements to form a comprehensive simulation system.

Simulator tests reproduce real conditions by loading and forcing the tested vehicle’s movement. Simplifications are sometimes implemented to simulate real conditions on the test rig. The rotating wheel of a vehicle makes an excellent example because it does not rotate in most test rigs. Yang et al. [7] presented a theoretical analysis using an improved “ring model” for a stationary and rotating wheel. The analysis was carried out for two cases: a loaded and unloaded wheel, evaluating the qualitative relationship between these conditions. The results were evaluated with a co-rotating and non-rotating reference frames model.

Crossing over an obstacle is a standard method of verifying the accuracy of simulator tests or mathematical models. A theoretical analysis of a wheel driving onto an obstacle and moving on the obstacle was presented by Weng and coauthors [8]. The analysis was carried out with ANSYS software. The paper presents the forces acting on the wheel during the wheel’s initial contact with the obstacle and when crossing over it. Hicks et al. [9] presented simulation and experimental test results of a quad bike passing over an obstacle, with particular emphasis on the dynamics of tire deformation. Martini et al. [10] described an experimental validation of a numerical multibody model of a forklift truck. For model validation purposes, the authors used vehicle vibrations registered when running over the obstacle. Naets et al. [11] described an obstacle identification method for various vehicles using simple acceleration sensors. The method uses vehicle parameters and real input data concerning the road’s profile in a single mass-spring-damper model. The importance of tires and their influence on the overall response of the system was emphasized by the team of Darguzis [12]. The authors presented a dynamic model for simulating the deflection and load of the tire as well as suspension load and displacement while the vehicle crosses over an obstacle.

The analysis of the active suspension system is also widely discussed due to its impact on the safety and comfort of driving, especially in heavy vehicles. Basaran [13] developed an analytical model of the active suspension system of a truck cabin. The system is controlled by an electromagnetic actuator, which, compared to a pneumatic system, is precise and operates with lower inertia.

Azrulhisham et al. [14], in their work, analyzed the fatigue of a suspension element based on data from the test track on the MTS 320 road simulator. The aim of their work was to compare the analytical calculations of fatigue with the results from the road simulator. The difference between theoretical analysis and road simulator results was 50%.

The authors of this paper present an analysis of the reproduction of a functional vehicle crossing over an obstacle with a known shape. The correctness of the actual conditions’ representation on the MTS 320 road simulator is evaluated. The evaluation is based on comparing the sensor responses from real runs with the data obtained from the road simulator. Moreover, the authors performed a mathematical analysis of the completed runs by developing the vehicle’s numerical model with two degrees of freedom. The model was used for comparing theoretical results with the data collected from the road and data generated by the road simulator.

The results presented in this paper are one of the stages of estimating the degree of vehicle wear during bench tests on a road simulator in comparison to the actual use of the vehicle by the user. The distance for reliable durability tests of finished products is 500,000 km (which is about 4 years of vehicle use). The overriding and utilitarian goal of the authors is to develop and validate a measurement methodology that will shorten the duration of durability tests on a road simulator to about 6 weeks. Correct determination of the parameters of the simulation test and the duration of the excitation, which would correspond to the specified runs of the semi-trailer, will radically shorten the test duration, reduce energy consumption, reduce the level of harmful impact on the natural environment and above all ensure the possibility of designing safe semi-trailer structures.

## 2. Tests

In the first stage of this work, road tests were carried out with use of a truck tractor with a semi-trailer. The tests consisted of multiple runs at different speeds over an obstacle of a defined shape. During the test runs, measurement data from sensors installed in selected places of the semi-trailer was recorded. The measurement setup and parameters of the semi-trailer are described in detail in Section 2.1.

Data obtained from the runs were used in the second stage of the tests, which were carried out on the MTS 320 stationary road simulator. Based on the recorded signals, and by performing an appropriate number of iterations, road profiles were developed. They consisted of signals controlling the hydraulic actuators, located under the wheels of the semi-trailer, in order to reproduce the shape of the obstacle used in the first stage of the tests. This procedure is described in detail in Section 2.2.

**Figure 1:**
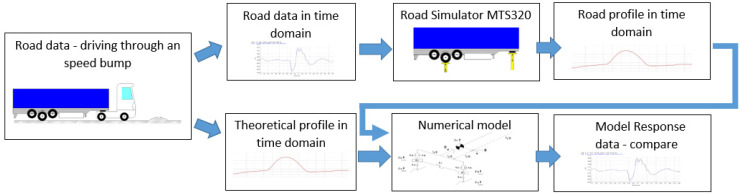
Flowchart of the tests. The upper part indicates operation steps during road simulator tests; the lower part shows the analysis procedure for the numerical model.

The final task was to develop a physical, mathematical and numerical model of the semi-trailer, taking into account its parameters and suspension characteristics, described in detail in Section 2.3. Road profiles (real, theoretical and developed on the MTS station) were the input data for the numerical model. The obtained results were compared and analyzed in the final sections of this paper. The test method described above is presented graphically in Figure 1.

### 2.1. Experimental Studies

A 13.8 m long NS3K triple-axle semi-trailer was used for the tests. The semi-trailer’s curb mass amounted to 6300 kg. The semi-trailer was equipped with a semi-active pneumatic suspension, controlled with a self-levelling valve, maintaining a constant travel height and adapting the pneumatic actuator’s pressure to the current load. The leveling valve is controlled mechanically by changing the ride height. When the ride height decreases, the valve increases the pressure in the pneumatic system, and when the ride height increases, the valve lowers the pressure. In this way, the pressure in the pneumatic cylinders is adjusted to the current load. The leveling valve also works while driving, so that the pressure in the system is constantly corrected. The damping system is equipped with a piston shock absorber with a constant damping characteristic, regardless of the axle load. A similar arrangement was analytically described by Krishnasamy et al. [15] where the pneumatic actuators were electronically controlled. The axle loads for a non-loaded semi-trailer amounted to 1.8 t per axle, while the fifth wheel coupling’s load was 2.5 t. The semi-trailer’s (technical) load capacity was 33 t. The axle load increased to 9 t for a loaded semi-trailer. The pressure in the pneumatic suspension system varies from 0 to 60 MPa.

The test was performed for a single-axle semi-trailer. For this purpose, the first and third axles were raised mechanically and locked. The semi-trailer moved only on the second axle with a fully functioning pneumatic suspension system. Such a system was tested in real conditions, then on a road simulator and in a numerical simulation. The test was carried out for a semi-trailer with one axle in order to analyze the influence of the ground on the suspension without the influence of the other axles on the system.

The main purpose of the tests was to determine the degree of correctness of mapping the passage through a given obstacle by the MTS durability test stand. The authors are aware of the fact that the introduced simplification of the number of axles in normal conditions is not possible. However, the procedure of blocking the operation of the other axles allowed for the isolation of the suspension system from the other axles and to better interpret the obtained results. The conducted research can be classified as preliminary research. The authors plan to repeat the research for the three-axis system in the future.

#### 2.1.1. Measuring Techniques

In order to collect the data during the test, the vehicle was equipped with nineteen sensors recording physical values in time. The sensors included acceleration, displacement and suspension system pressure sensors, and strain gauges. The details of the sensors and their ranges are presented below:

- 1 MEMS capacitive accelerometer (ASC model 5521-050) with measurement in three axes (3 channels), a frequency response range from 0 [Hz] to 4000 [kHz] (±3 dB), a measuring range of 30 g and a sensitivity of 90 mV/g;
- 9 MEMS capacitive accelerometers (ASC model 4421MF), with a frequency response range from 0 Hz to 7 kHz (±3 dB), a measuring range of 30 g and a sensitivity of 90 mV/g;
- 6 displacement sensors (Waycon type SX50-625-1R-KA02-HG), with a measuring range of 625 mm, linearity up to ±0.02% of full scale and a resolution of 10 [imp/mm];
- 2 half-bridge strain gauges 120 Ω, one active and one passive (KYOWA model KFGS-10-120-C1-11), a gauge factor of 2.13 ± 1.0%, a bridge excitation of 2.5 V and a bridge factor of 2;
- 1 pressure sensor (HBM type P8AP/20), with a measuring range of 0–20 bar, a rated sensitivity of 2 ± 2% mV/V and a natural frequency of 16 kHz.

Quantum HBM series measurement cards were used for data acquisition. The data were recorded with a CX22 data recorder. The CX22 card from HBM enables simultaneous on-line data acquisition from various types of measurement sensors. The measurement frequency for each channel amounted to 300 Hz. During the measurements, the measured signals were filtered using a Bessel anti-aliasing filter. This type of filter is used in the HBM data recorder as the default filter.

#### 2.1.2. Distribution of Sensors on the Vehicle

**Figure 2:**
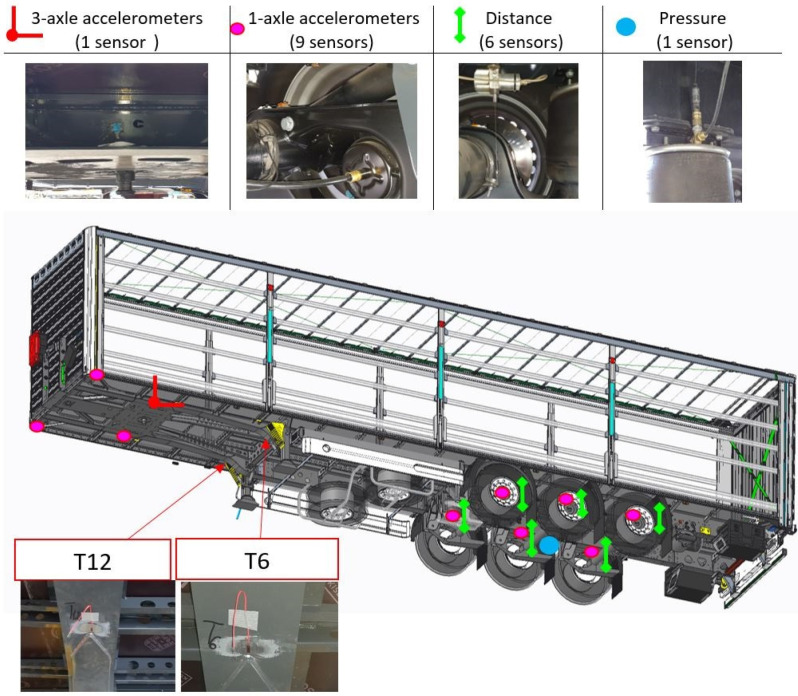
Locations of the acceleration, pressure and displacement sensors on the semi-trailer.

When the vehicle was crossing over the obstacle, the sensors on the semi-trailer were arranged to enable the road simulator’s operational control. Since the inputs from hydraulic actuators are located under the wheels when the road profile is generated on the simulator, the authors decided to place the sensors near the axle. The sensors were placed as close to the vehicle’s wheels as possible to control each wheel independently. The measurement sensors’ operation direction was the same as that of the input generated during the durability tests. The locations of the installed sensors are shown in Figure 2. Since the vehicle was equipped with a pneumatic suspension system, pressure sensors were installed to control pressure change depending on the load. An active pneumatic suspension system aims to maintain constant ride height regardless of the load, adapting the pressure to the current load. Moreover, displacement and acceleration sensors were installed on each axle. The frame’s front (coupling) part was controlled by five accelerometers and two strain gauges located at the frame cross-section change area, where the most significant stress changes were expected due to the vehicle frame’s torsional motion.

#### 2.1.3. Description of the Obstacle

**Figure 3:**
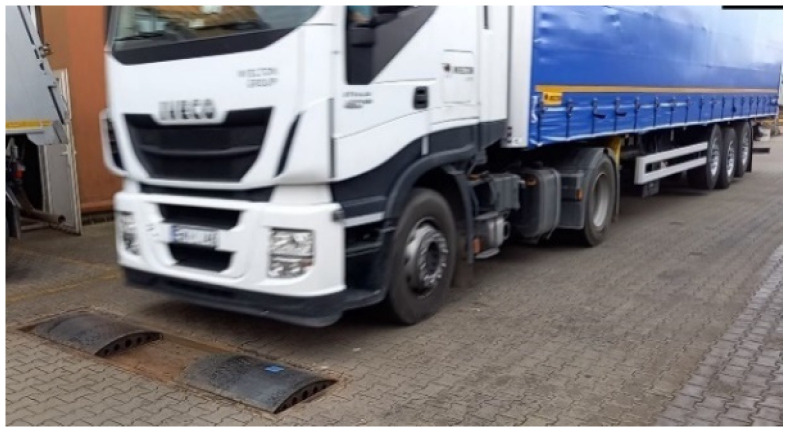
Truck combination during the test.

Crossing over the obstacle was performed with a biaxial road tractor and triaxial semi-trailer with the first and third axle lifted (Figure 3). The four driving speeds in the tests amounted to 14, 18, 34 and 39 km/h. When selecting the speed of crossing an obstacle, the key aspect was to ensure that the wheels did not lose contact with the ground. Moreover, for most physical traffic calming measures the recommended speeds are between 20–30 km/h.

**Figure 4:**
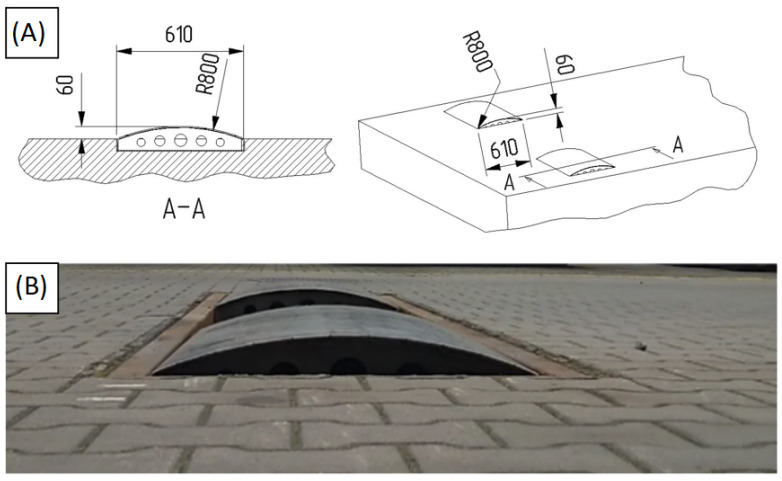
Speed bump used for the test: (**A**) dimensions and (**B**) view of the speed bump.

An artificial obstacle (speed bump) shaped as a circular arc section with R = 0.8 m radius was used for the tests. The obstacle was placed on a paved road in one line for two wheels so that one axle’s wheels approached the obstacle simultaneously. In Figure 3, the track combination during the test is depicted, whereas Figure 4 shows the obstacle’s cross-section. The obstacle’s height at the highest point was 0.06 m. The test run was performed as described in the work of Czarnuch et al. [16].

The data were collected in the form of time signals from the sensors installed on the vehicle when the unloaded semi-trailer, moving on one axle, was crossing over the obstacle at different speeds. The collected data applied to acceleration, displacement and suspension system pressure.

### 2.2. Determination of the Run Data on the Road Simulator for the Tested Cases

**Figure 5:**
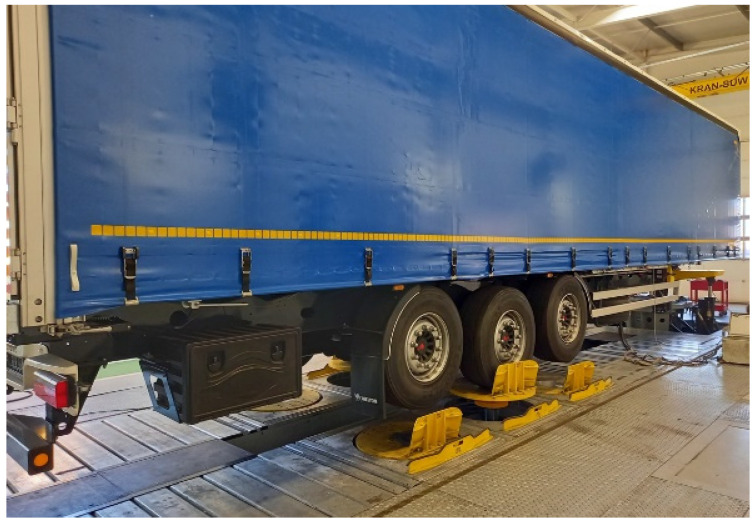
Vehicle installed on the MTS road simulator.

After the field tests, the vehicle was placed in the MTS 320 road simulator test rig, as visible in Figure 5. A detailed description of the road simulator used for the tests was presented by Stembalski at al. [17]. It is a durability testing rig for triaxial vehicles with the coupling part, simulating a road tractor. The test rig parameters were adapted to the tested product’s dimensions and weight. The semi-trailer was supported at the fifth wheel coupling area and at one axle at the back. The first and the last axles were lifted and mechanically secured to imitate the coupled unit’s real run over an obstacle.

The recorded measurement data from the sensors are processed using the MTS RPC Pro software and filtered in a range from 0.6 to 50 Hz.

After preprocessing the data, the next step is System Modeling. The purpose of the System Model is to define the relationship between inputs and outputs. The input signals are the actuators of the control station designated as “Drive”. The output signals are the data obtained from the sensors installed on the vehicle designated as “Response”.

**Figure 6:**
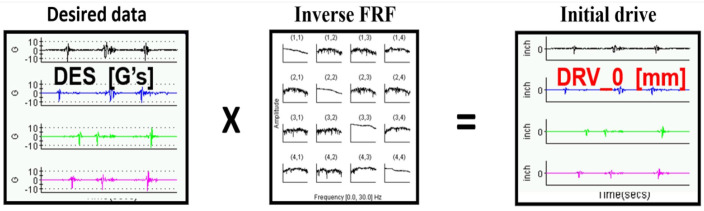
Model of the system.

The System Model is a frequency response function (FRF). It shows the relationship between the output and the input over the entire band of acquired frequencies. In other words, for a given input, it is possible to specify an output. The “Drive” signal of displacement of actuators is determined by multiplying the signals received from the road by the inverse of the FRF matrix. The scheme of the procedure is shown in Figure 6. A more detailed description of the model creation and further steps at the MTS station are described in detail in our other paper [18].

**Figure 7:**
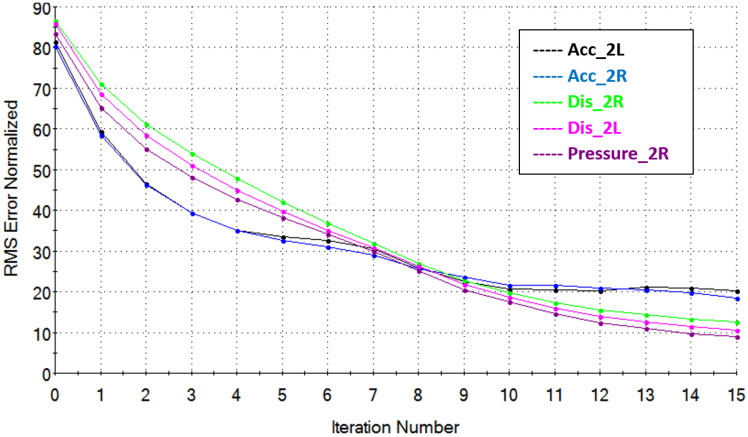
RMS of signal reconstruction vs. number of iterations for second axis during test runs over the defined obstacle.

After determining the System Model using the MTS RPC Pro software, signals controlling the movements of the hydraulic cylinders are optimized in order to recreate the signals from sensors collected during the road tests as accurately as possible. This is the stage of signal reconstruction. The signal waveforms are reconstructed in several (over a dozen) iterative steps. After each iteration step, the signal reconstruction level as well as its frequency level and reconstruction error are controlled by taking into account the root mean square (RMS) of the expected signal to the desired one. An example of the signal reconstruction result as a function of the number of performed iterations is shown in Figure 7.

Based on the data collected from the real runs, the control signals for the test rig’s actuators were reproduced for each tested case. Because of the large volume of information, only representative data for the right wheel of the vehicle’s second axle were selected for the presented paper (Figure 7).

Then, simulation iterations were performed to reach the best convergence of the simulation results with the actual ride. Comparing the RMS value of the measured real signal with the signal obtained from the simulation at the MTS, convergence ranging from 94% to 99% was achieved.

### 2.3. Theoretical Model

**Figure 8:**
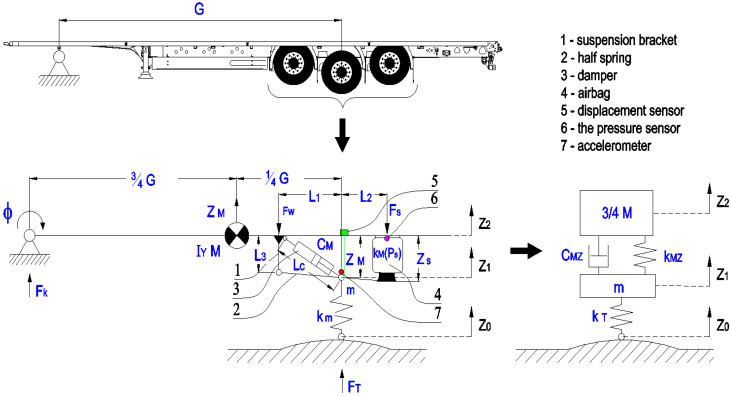
Illustrative model of single-axle semi-trailer with two degrees of freedom.

The first run with an unloaded single-axle semi-trailer was used for numerical analysis of the vehicle’s dynamics when crossing over an obstacle. The run was performed for the vehicle crossing over an identical-shaped obstacle with the right and left wheel. The semi-trailer’s coupling part on the road tractor did not make any vertical movements when the wheels were crossing over the obstacle. The coupling part is 7.7 m away from the sprung part. Considering the above, the numerical model was simplified to a model with two degrees of freedom, e.g., as in M. Jamali’s paper [19]. The kinematic diagram of the semi-trailer and the numerical model are shown in Figure 8. Vibrations were included in the following degrees of freedom:

- Z_2_ vertical displacement of the vehicle as M;
- Z_1_ vertical displacement of m, in the middle of the vehicle’s wheel.

Figure 8 shows the position of the acceleration, displacement and pressure sensors when crossing over an obstacle. The suspension system was simplified to a flat system where the force from the wheel is distributed between the suspension bracket 1 (F_W_) and the suspension air spring 4 (F_S_). As it was described in our earlier paper [20], the distribution of the forces results from the suspension kinematics and the L_1_ and L_2_ ratio of the semi-spring dimensions. The Z_M_ axle displacement against the frame results from the Z_S_ suspension air spring’s deflection. The relationship is described with the following Equation (1):

(1) $$Z_{s}+\Delta Z_{S}=\frac{L_{1}+L_{2}}{L_{1}}\cdot \left(Z_{2}-Z_{1}\right).$$

where:

$Z_{S}$—height of the air springs

$L_{1}$—semi-spring’s length, bracket-axle

$L_{2}$—semi-spring’s length, axle-air spring

$Z_{1}$—displacement of unsprung mass

$Z_{2}$—displacement of sprung mass

A suspension airbag ensures the suspension’s constant static height by pressure change; it requires considering stiffness change and non-linear effects. In this paper, a system with pneumatic lines of small diameter ϕ10 mm was analyzed. As shown by Chen et al. [21], such a diameter of hoses does not affect the dynamic characteristics of the suspension between the axle system.

The stiffness of the elastic system was selected on the basis of the static load on the axle. The characteristics were linearized with the gas air spring operation range, as presented in the studies of Moheyeldein et al. [22]. A substitute rigidity of the springing system was determined based on the suspension kinematics using the suspension manufacturer’s catalogue data [23]. The relationship is described with the following Equation (2).

(2) $$k_{MZ}=A_{E}\cdot \frac{dP_{S}}{d_{Zs}}$$

where:

$k_{MZ}$—suspension stiffness coefficient for the set pressure in the air spring

$A_{E}$—effective cross section area of the air spring

$P_{E}$—pressure in the air spring

$Z_{S}$—height of the air springs

Tire stiffness k_m_ was determined based on the manufacturer’s catalogue data [24]. However, since tires have low damping values, their impact is often neglected in analyses, assuming that only the shock absorber is responsible for damping in the system. Such an approach was used in the studies of Ashtekar [25] and Brethiye [26].

A shock absorber with the damping coefficient C_M_ was used. The shock absorber in the system is mounted at an angle from the axle to the suspension bracket. The shock absorber’s elongation correlation with the changing axle displacement to the frame is described by Equation (3).

(3) $$L_{C}+\Delta L_{C}=\sqrt{{L_{1}}^{2}+(Z_{2}-{Z_{1})}^{2}}$$

where:

$L_{C}$—initial length of the shock absorber

Substitute damping C_MZ_ (physical model—Figure 8), taking into account the shock absorber’s angular position, is described by relationship (4).

(4) $$C_{MZ}=C_{M}\cdot \frac{\sin\left(\tan^{-1}\left(\frac{Z_{2}-Z_{1}}{L_{1}}\right)\right)}{\cos\left(\tan^{-1}\left(\frac{L_{1}}{Z_{2}-Z_{1}}\right)\right)}$$

where:

$C_{MZ}$—substitute damping

$C_{M}$—shock absorber’s damping coefficient

A mathematical model developed based on a semi-trailer’s simplified physical model can be described with Equations (5) and (6):

(5) $$\frac{3}{4}M\cdot {\ddot{Z}}_{2}={-k}_{MZ}\cdot \left(Z_{2}-Z_{1}\right)-C_{MZ}\cdot \dot{{(Z}_{2}}-\dot{Z_{1}})$$

where:

M—sprung mass

¾ sprung mass (M) results from vehicle mass sprung by vehicle rear suspension. The rest is carried by the truck saddle.

(6) $$m\cdot {\ddot{Z}}_{1}=k_{MZ}\cdot \left(Z_{2}-Z_{1}\right)+C_{MZ}\cdot \dot{{(Z}_{2}}-\dot{Z_{1}})-k_{T}\cdot \left(Z_{1}-Z_{0}\right)$$

where:

m—unsprung mass

$k_{T}$—tires stiffness coefficient

$Z_{0}$—road profiles

The semi-trailer’s simulation model was developed in MATLAB, with the Simulink environment. The values of its parameters are summarized in Table 1.

The displacement signal for the following two cases was adopted as the Z_0_ input. The signal generated by the MTS road simulator based on the semi-trailer’s real crossing over an obstacle was the input in the first case; in the other case, a theoretical crossing over an obstacle in time constituted the second input signal. The output signals were the non-sprung weight acceleration, non-sprung weight displacement against the sprung weight and pressure in the suspension air spring. They were the values recorded during the tests.

## 3. Results

Speed bump driving tests were carried out for several speeds: 14 km/h, 18 km/h, 34 km/h and 39 km/h. The tests were carried out for an empty, unladen set, with one axle of the semi-trailer passing over a speed bump (the other two axles were raised). The data collected from the road tests was subsequently used to develop road profiles for the MTS station.

### 3.1. Results from the MTS 320 Test Rig

The first stage of the test involved generating a signal under the vehicle’s wheels and coupling part of the semi-trailer on the MTS 320 road simulator. The inputs under the vehicle wheels were reproduced based on the data collected during the semi-trailer’s actual runs over an obstacle.

**Figure 9:**
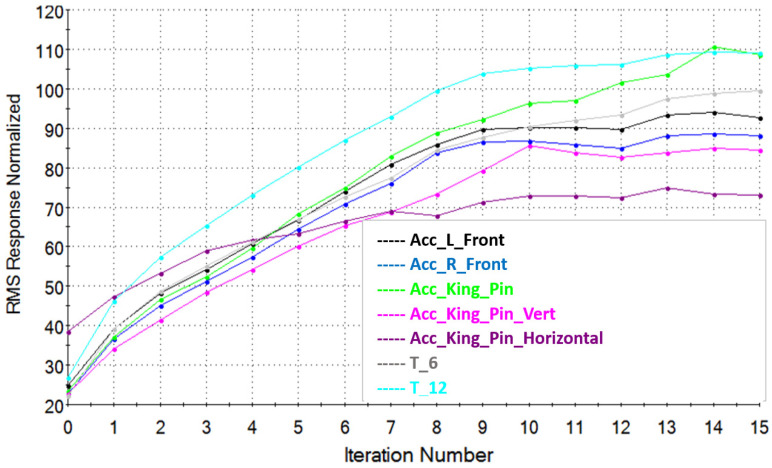
Normalized RMS of the recorded real signal with the simulation signal for each iteration. Acc_L_Front—acceleration in the front left corner in the vertical direction; Acc_R_Front—acceleration in the front right corner in the vertical direction; Acc_King_Pin—acceleration in the vicinity of the king pin in the vertical direction on the left side; Acc_King_Pin_Vert—acceleration near the king pin in the vertical direction on the right side of the trailer; Acc_King_Pin_Horizontal—acceleration near the king pin in the longitudinal direction on the right side of the trailer; T_6—tension from the strain gauge in the area of the gooseneck, left side; T_6—tension from the strain gauge in the area of the gooseneck, right side.

**Figure 10:**
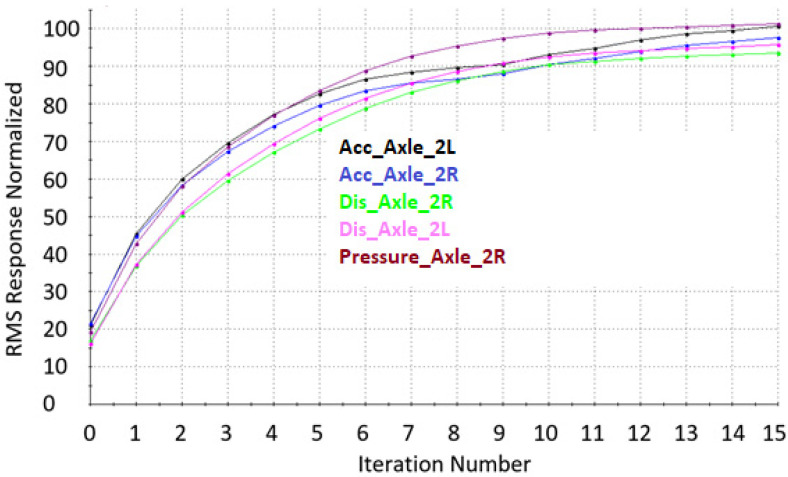
Normalized RMS of the recorded real signal with the simulation signal for each iteration. Acc_Axle_2L—acceleration on the 2nd axle right side; Acc_Axle_2R—acceleration on the 2nd axle left side; Dis_Axle_2R—displacement of the 2nd axle on the right side; Dis_Axle_2L—displacement of the 2nd axle on the left side; Pressure _Axle_2R—pressure in the suspension cushion on the 2nd axle on the right side.

Figure 9 shows the convergence of Normalized RMS response results for accelerometers located in the front part of the semi-trailer frame and strain gauges T6 and T12, located in the place of changing the cross-section of the main beam. Sample convergences of the results after a dozen or so iterations obtained on the acceleration, displacement and pressure sensors installed on the semi-trailer’s second axle are shown in Figure 10. 

The analysis of the waveforms for individual iterations of the Normalized RMS percentage value indicates that the sensors that were placed on the axles in the place of the excitation generated by the hydraulic cylinders on the MTS test stand are characterized by better convergence to 100% of the signals recorded during the road tests. The rest of the signals, especially those coming from the accelerometers located in the front part of the trailer, are characterized by a smaller convergence of around 100% and a greater spread between 70–110%. This is due to the fact that in the front part of the semi-trailer there is only one actuator under the king pin and a second smaller one responsible for simulating the side inclinations of the semi-trailer. In addition, the connection of the king pin with the saddle is characterized by significant looseness, which causes a problem with mapping the signals coming from the sensors in the vicinity of the saddle.

For this reason, in further analysis the authors decided to take into account the signals coming from the sensors placed on the axis.

**Figure 11:**
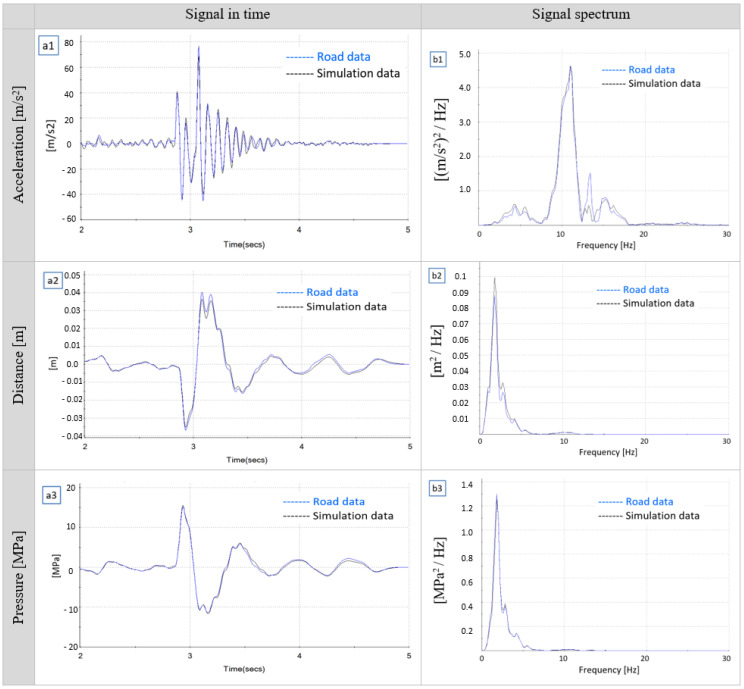
Representation of the real signal in the time and frequency domain for acceleration, displacement and pressure on the MTS road simulator.

The left side of the diagram in Figure 11(a1–a3) compares the reference signals obtained in the real tests with the simulation signals for the selected speed of 18 km/h. On the right-hand side of the diagram, Figure 11(b1–b3), the acceleration, displacement and pressure signals are compared in the frequency domain. Analyzing the diagrams, it can be stated that the values of the measurement signals obtained in the simulations and recorded during the real runs, in great majority, correlate in terms of character and value, which confirms the usability of the applied simulation model.

**Figure 12:**
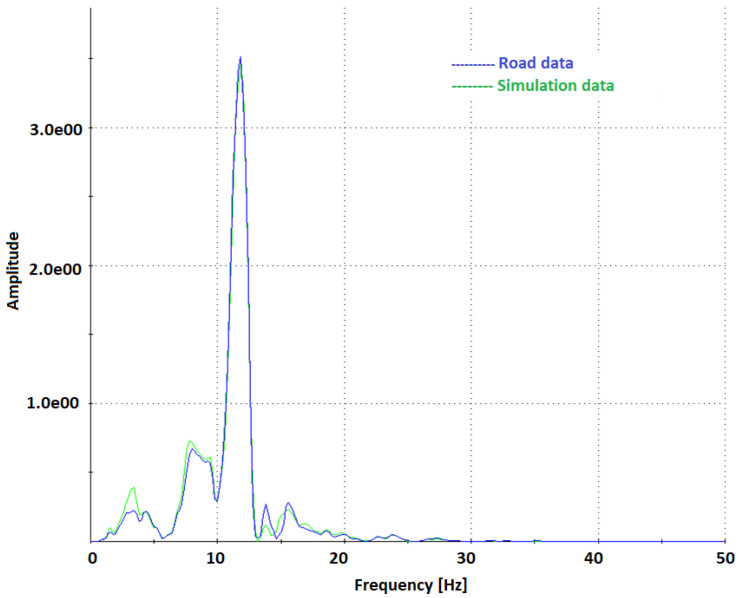
Comparison of acceleration spectra for road data and MTS simulation for travel speed of 14 km/h.

Interestingly, in Figure 11(b1), for the accelerometer at a frequency of approximately 13 Hz a significant discrepancy in amplitude between the signal measured on the road and the one reproduced by the MTS station can be noticed. It may result from the difference between the road tests and MTS simulation conditions, namely the distribution of forces. The MTS station does not generate longitudinal forces (thus it is impossible to simulate braking and acceleration of the vehicle). In contrary, during the real runs, when driving over the speed bump, a horizontal force component is also present. Moreover, at low speeds, such differences were not noticed. Therefore, it should be additionally analyzed whether during the real runs there was any loss of contact between the tire and the road surface. Figure 12 shows the spectrum for the accelerometer for a lower travel speed of 14 km/h. It clearly confirms that at lower speeds, for the frequency of 13 Hz, no discrepancies in the amplitudes of road data and MTS simulation results were observed.

**Figure 13:**
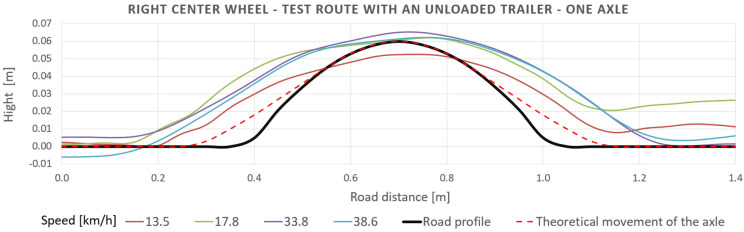
Drive data under the right wheel in comparison to the real shape of the obstacle (speed bump).

Figure 13 shows the actuator’s vertical displacement signal as a function of the vehicle’s displacement (lengthwise displacement) under the right wheel, for which the signals from the road were reproduced on the road simulator. The results were compared with the obstacle’s theoretical shape for different driving speeds. As the input signal, the generated road profile has a similar height as the obstacle’s theoretical shape, with up to 13% error. Still, the profile is wider than the assumed theoretical shape. It results from the radius of the wheel and a shift of the tire’s contact point with the obstacle in front of the axle.

### 3.2. Results for the Numerical Model in Simulink

**Figure 14:**
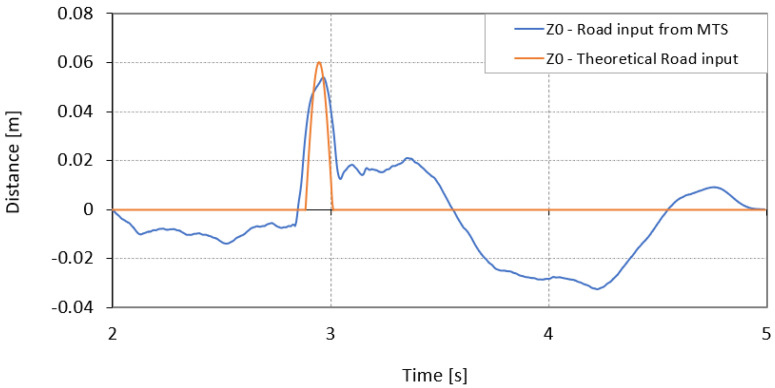
Time signals as road inputs for Simulink model.

The next step involved an analysis of the results for a theoretical model developed in Simulink. The signal from the semi-trailer’s right wheel for the speed of 18 [km/h] was used to analyze the results. The first analysis compared the responses for the numerical model with the responses for the real runs, where the signal generated by the test rig was the input. The signal from the road simulator was selected as the input signal under the wheel because the actuator only moved vertically. Such a signal does not generate transverse forces, which are not considered in the numerical model. The next stage involved generating the numerical model’s response when the road profile is a theoretical shape of the obstacle that is crossed over. In Figure 14, the time signal of the actuator’s vertical displacement generated on the simulator compared to the theoretical signal describing the road shape is depicted. The vertical displacement of the actuator in the first part is close to the theoretical one in terms of shape and amplitude up to 2.9 s of the test, when the wheel runs into an obstacle. The difference lies in the time of leaving the obstacle when the actuator’s displacement does not return to the theoretical position’s level. It is the actuator’s vertical movement for which the behaviour of a vehicle moving on a simulator is reproduced.

**Figure 15:**
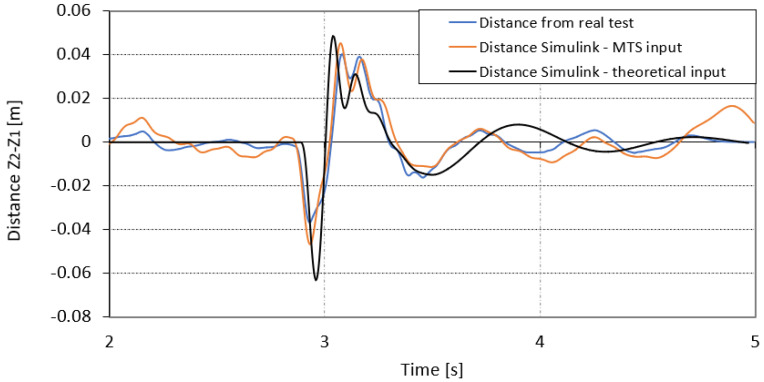
Z_2_–Z_1_ amplitude response from Simulink calculations for theoretical, MTS and real run displacement conditions.

Figure 15 shows the displacement of the unsprung mass in relation to the sprung mass (Z2–Z1) for two numerical simulations displacements registered during the road test. The numerical simulation was performed for two input signals under the wheel:

- The first was the theoretical road shape signal for which the displacement signal was marked in black;
- The second was the displacement signal marked in orange, generated for the input signal under the wheel from the MTS test rig.

The signals from the numerical simulation were compared to the displacement signal recorded during the test. The nature of the numerical signals reflect the displacement recorded in real conditions. In the simulation, the amplitude of the signal generated on the basis of the theoretical road shape presents higher level of displacement (approximately −0.06 m) compared to the displacement recorded during the real drive (approximately −0.035 m). The displacement generated on the basis of the MTS input signal is closer to reality and amounts to approximately −0.42 m.

**Figure 16:**
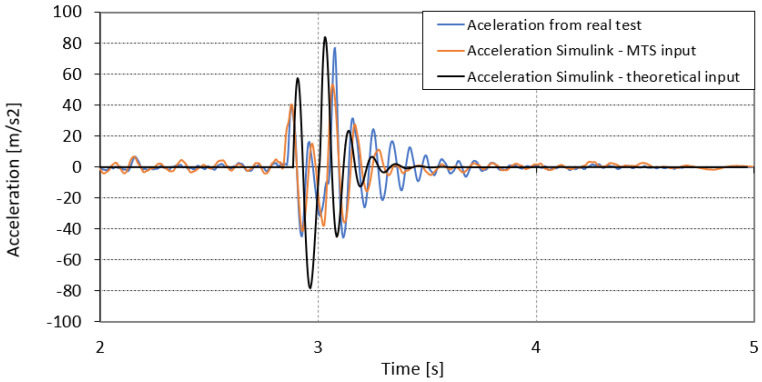
Acceleration amplitude response from real run and Simulink calculations for theoretical and MTS displacement conditions.

The acceleration of the unsprung mass over time for numerical simulations and actual accelerations recorded during the test are presented in Figure 16. The nature and magnitude of the acceleration signal for the numerical simulation based on the input signal from MTS was mapped similarly to the acceleration recorded during the road tests. The acceleration signal mapped on the basis of the signal from the theoretical shape of the road differs from the real signal in terms of frequency and time shift. The acceleration recorded during the approach to the obstacle in 2.9 s of the test was correctly mapped on the basis of the signal from the road simulator.

**Figure 17:**
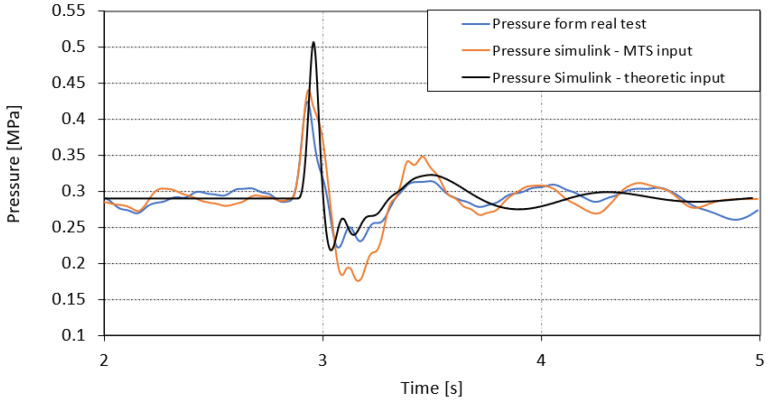
An air spring pressure response amplitude for the real test and the Simulink calculations for theoretical and MTS displacement conditions.

In Figure 17, the change in pressure in the suspension cushion while driving over an obstacle is presented. The results of numerical analyses and the real test were also compared. The pressure signal determined on the basis of the input data from the MTS station was mapped in a very similar way to the data recorded during the test runs. The difference is noticeable after the wheel leaves the obstacle in 3.2 s of the test. In real conditions, the pressure drops to 0.22 MPa, whereas for the numerical simulation using the signal from the simulator, the pressure drops to about 0.17 MPa. The signal generated from the theoretical shape of the obstacle generates a higher pressure while approaching the obstacle in 2.9 s of the test; the difference is about 0.08 MPa.

In order to estimate the error of the reproduced signal, for each waveform the RMS was calculated. The percentage difference in the signals is summarized in Table 2. The time waveforms and the estimated RMS error of the signals indicate a 13% better representation of the road conditions for using the road profile that was reproduced by the road simulator. Analysis employing the theoretical profile as the input signal generates up to 24% reproduction error of the signals recorded during the real run, versus the signals obtained in the simulations. Therefore, other factors need to be taken into account, such as rolling resistance, earlier driving onto and leaving the obstacle and taking into account the tire’s rolling deformation.

For the case when the signal generated by the road simulator is the input signal in the numerical model, the signal reproduction error based on the RMS difference decreases to 11%. It confirms that the factors that were not considered in the numerical model were included in the MTS 320 road simulator. The input generated on the actuator under the wheel acts only vertically, whereas the transverse and longitudinal forces are neglected in this case. Pazooki et al. [27] proposed a dynamic model including an adaptive footprint radial tire model. For such a model, the authors confirm the convergence of the obtained results with the measured accelerations, ranging from 4 to 14% of the computed and measured data’s RMS value.

This paper validated the model for the three physical values: acceleration, displacement and pressure. It enables controlling the physically measured values and analyzing the impact of the suspension’s variable parameters on the system’s responses.

## 4. Discussion

The authors verified the recorded run numerically by developing a vehicle model described with motion equations. In the referenced case, when the signal generated by the road simulator is the input signal in the numerical model, the signal reproduction error based on the RMS difference ranges from 9 to 11%. This is because the signal from the road simulator, as a signal reproducing road displacement, is only a vertical input (actuator’s vertical displacement), and hence it can be successfully used as an input for the theoretical analyses of similar structures. However, for a theoretical input signal describing the obstacle’s shape, the profile representation error increases to 24%, suggesting that the road’s theoretical shape as the input profile does not ensure the correct representation of road conditions.

The results obtained constitute excellent input data as the boundary conditions in FEM computer simulations for fatigue analyses. The recorded data present the displacement covered in time and the forces acting both in the suspension components and on the structure.

The vehicle’s numerical model can also be used for analyzing and simulating variable road conditions, including the possibility of changing the suspension parameters. The model presented in this paper was validated for the primary parameters describing the suspension. It will enable further analysis of how the suspension system’s parameters impact its response and optimization.

The case of the obstacle crossing, described in this paper, is one of the stages of validation of the road simulator stand. In the case of tests on road simulators, it is important to correctly reproduce real road conditions and determine the main factors affecting the correctness of their mapping. In our work, we analyzed simple passes through the obstacle in order to assess the quality of the road mapping by the road simulator. We also analyzed the simple case of the vehicle moving on one axis to simplify the model as much as possible. Both were aimed at verification of the results obtained from the simulation in comparison to those registered during the test runs of the real object. In this way, it was possible to more accurately estimate at what stage of the analysis an error is made and what it may result from.

As it was presented, the control signal under the wheels of the vehicle generated on the road simulator is not the same as the shape of the road profile. This is related to the shape and stiffness of the tire, also demonstrated by Dargužis et al. [12]. The authors built a tire model to simulate a step-shaped obstacle approach, thus taking into account the additional longitudinal forces acting through the tire on the suspension system.

The novelty of the presented work is the determination of possible sources of error generated during analytical simulation compared to real tests conducted using road simulator stands. The purpose of tests on road simulators is to determine the durability of the vehicle and compare it with reality. Determining the error at each stage of the test is crucial to determine the estimated fatigue and life cycle of the vehicle at the end. Referring to the work of Azrulhisham et al. [14], the analytically calculated fatigue strength of the car steering knuckle system in relation to the fatigue determined during the simulation on the road simulator differed by 50%, which is a relatively large difference. The improvement of the results is possible only through a thorough analysis of the individual stages of the methodology used.

The generated ideal shape of the obstacle in the numerical model results in an error of 24%. It can therefore be assumed that the introduction of an obstacle of the same shape in the simulation will generate at least the same error at the MTS station.

The authors are aware that the tests conducted for only one axle are a certain simplification. In fact, during durability tests, the semi-trailer most often works on two or three axles. Also, the numerical model does not take into account, among others, the aspects related to the rotation of the wheel when crossing an obstacle, the friction associated with the ground or the feedback and mutual interaction of the axles with respect to each other. In the future, the authors intend to perform an analytical model that takes into account all three axles in a large-size vehicle.

## 5. Conclusions

The results presented in this paper confirm the validity of the adopted research method aimed to represent road conditions on a road simulator. Furthermore, the authors prove the convergence of the results for real runs with those reproduced on the simulator. Based on the RMS value of the reproduced signals to the collected signals, the error ranges from 1 to 6%. This is an excellent result for tests on large objects, such as semi-trailers, mainly because the data are reproduced from a dozen or so sensors in one simulation period.

It was proven, on the example of a simple passage, that when using data from a road simulator, the error at the signal mapping stage is very small (from 1–6%), while in the case of an numerical approach using the theoretical shape of the obstacle, the reconstruction error reaches 24%. Therefore, it seems to be a better solution to analyze the signals from the sensors located on the tested object and, on this basis, develop the excitation, as proposed in this work. Despite many different types of sensors located on the axles and in the front part of the semi-trailer’s frame, the best compatibility of mapping signals coming from the road was obtained for sensors located directly on the analyzed axle. 

## Figures and Tables

**Table 1:** Parameters of the mathematical model.

Description	Symbol	Value	Units
Sprung mass	M	5600	[kg]
Non-sprung mass	m	700	[kg]
Suspension stiffness coefficient for the set pressure in the air spring	k_MZ_	84,500	[N·s/m]
Shock absorber’s damping coefficient—rebound	C_M_ (R)	46,000	[N·s/m]
Shock absorber’s damping coefficient—compression	C_M_ (C)	14,000	[N·s/m]
Tires stiffness coefficient	k_T_	1,125,000	[N·s/m]
Pin-axle distance	G	7.70	[m]
Set ride height	Z_M_	0.25	[m]
Semi-spring’s length, bracket-axle	L_1_	0.5	[m]
Semi-spring’s length, axle-air spring	L_2_	0.35	[m]
Height of the suspension bracket	L_3_	0.25	[m]
Initial length of the shock absorber	L_C_	0.49	[m]
Tires	385/65/R 22.5	5600	[kg]

**Table 2:** Signal reproduction by the MTS road simulator and analytical model in Simulink based on the RMS value.

Signal Type	MTS 320 Road Simulator	Simulink Input Signal from the MTS	Simulink—Obstacle’s Theoretical Signal as the Input Signal
RMS error for Acceleration	1%	11%	24%
RMS error for Displacement	6%	8%	11%
RMS error for pressure	3%	9%	12%

## Data Availability

Not applicable.
